# Supplementary material for: Defense Responses Stimulated by Bacillus subtilis NCD-2 Through Salicylate- and Jasmonate-Dependent Signaling Pathways Protect Cotton Against Verticillium Wilt
Source: Int J Mol Sci. 2025 Mar 25;26(7):2987. doi: 10.3390/ijms26072987 (PMC11988308; doi:10.3390/ijms26072987)
Supplement: Supplementary file 1 [file ijms-26-02987-s001.zip › Supplemrntary Figures .pdf]

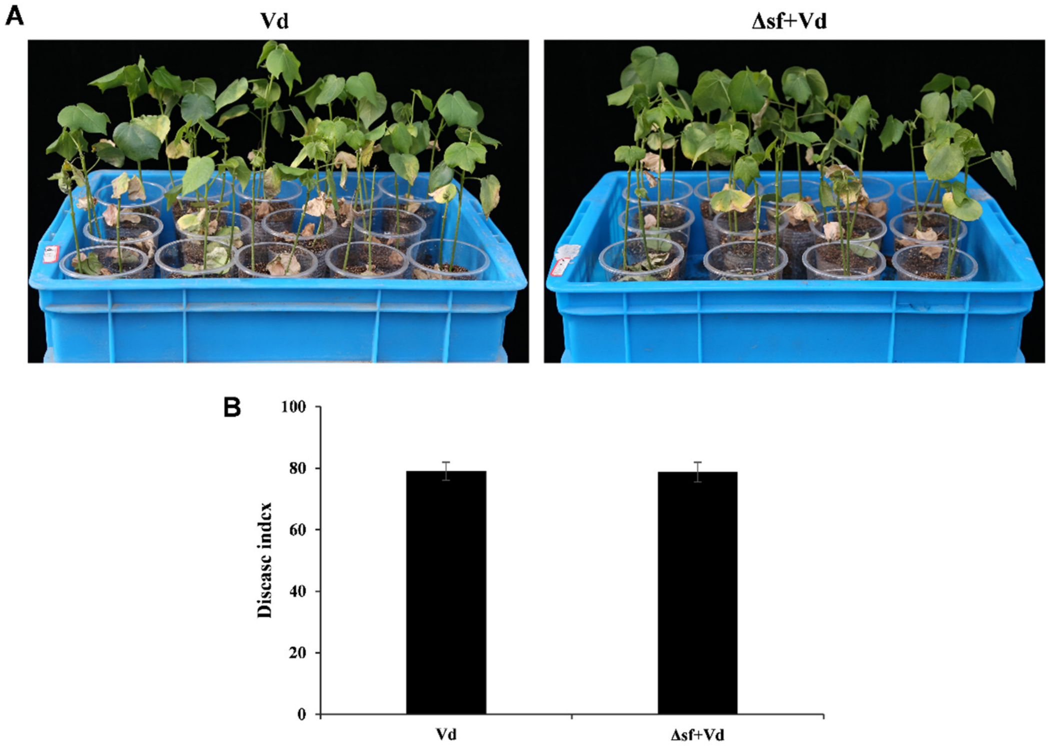

Figure S1 Disease symptoms of Δsf strains pre-treated plants upon *V. dahliae* inoculation. **(A)** Disease symptoms of Δsf suspension pre-treated and CK plants after inoculation with *V. dahliae* strain wx-1. Photographs were taken 25 days post wx-1 inoculation. **(B)** The disease index of the Δ*sf* pre-treated and CK plants. The values are the means ± SE, n = 3.

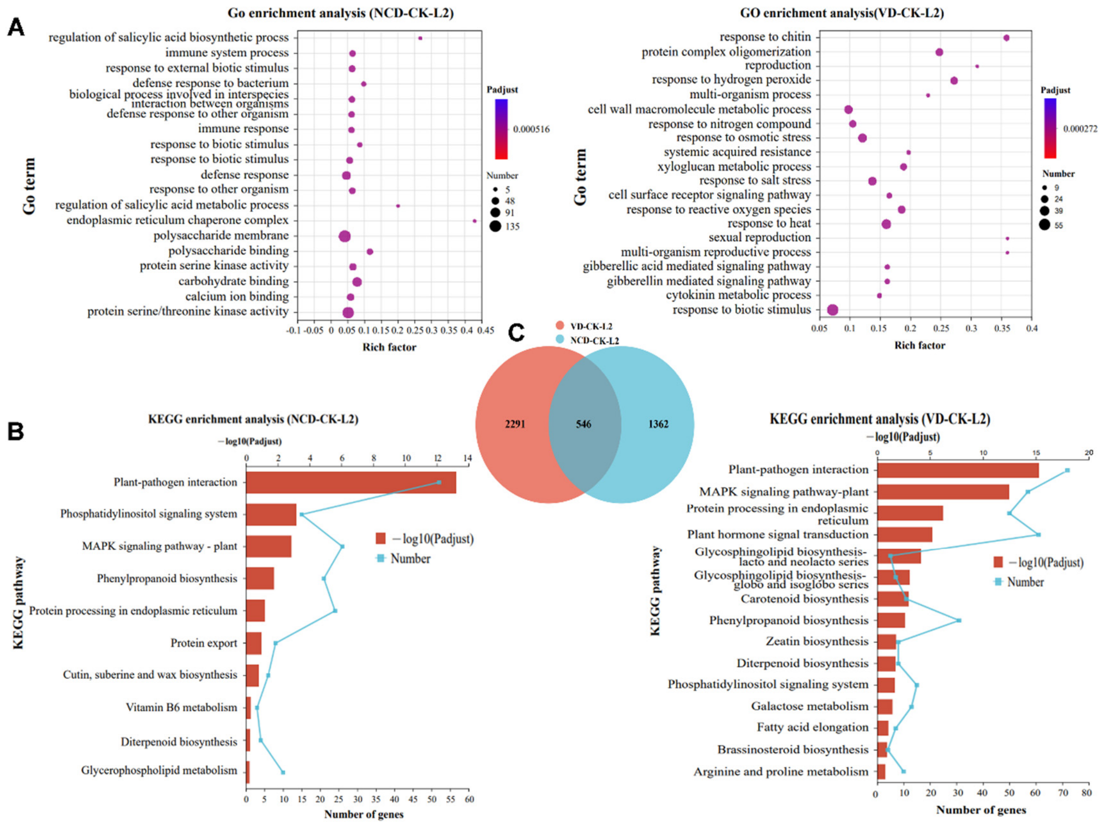

Figure S2 Comparative analysis of the DEGs resulted by NCD-2 treatment with *V. dahliae* treatment in the cotton leaves. **(A)** GO enrichment analysis of the DEGs in NCD-2 or *V. dahliae*-treated cotton leaves. **(B)** KEGG pathway enrichment analysis of the DEGs in NCD-2 or *V. dahliae*-treated cotton leaves. **(C)** The overlap of DEG abundance between the NCD-2 and *V. dahliae* treatment is presented in a Venn diagram.

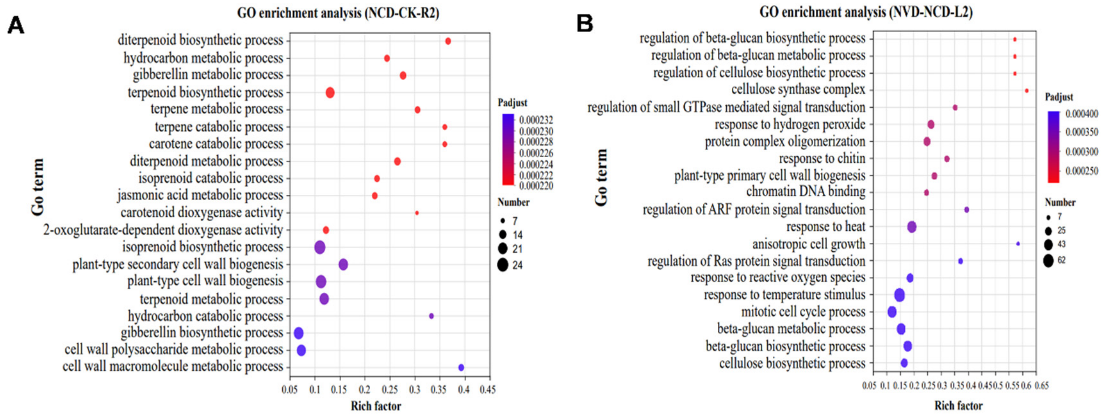

Figure S3 GO enrichment analysis of the DEGs in different treatment. **(A)** GO enrichment analysis of the DEGs in NCD-2-treated cotton roots. DGEs screened by more stringent criteria were used for the GO analysis (Padjust < 0.05). **(B)** GO enrichment analysis of the DEGs in the NVD/NCD group cotton leaves.

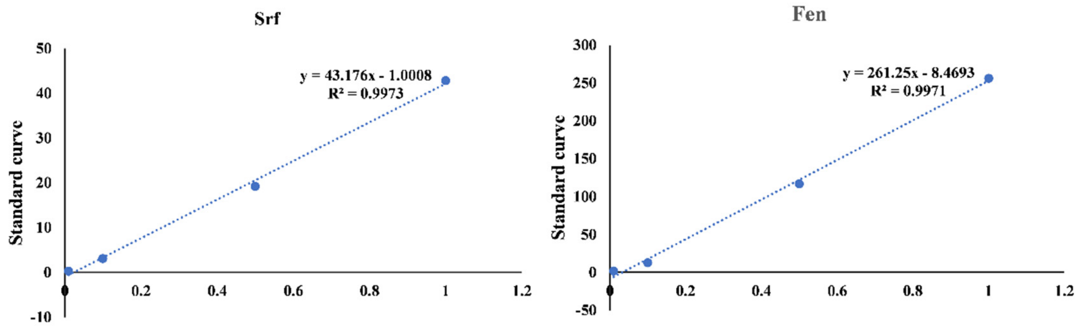

Figure S4 Standard curves of surfactin and fengycins drawn through FPLC method.

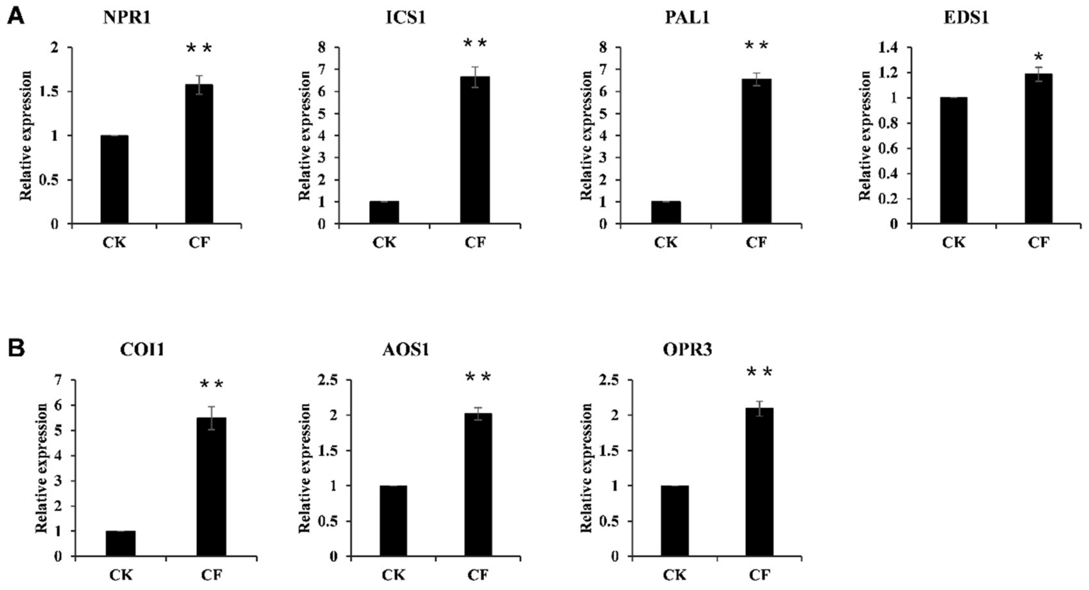

Figure S5 Lipopeptides produced by *B. subtilis* NCD-2 activates the SA and JA biosynthesis and signaling pathways in cotton. **(A)** Expression levels of SA signaling pathway genes in the cotton roots upon CF and CK treatment. Values represent the means ± SE for three biological replicates (**P* < 0.05, ** *P*< 0.01, Student's *t*-test). **(B)** The transcript levels of JA signal transduction pathway genes in the cotton roots upon CF and CK treatment. Values represent the means ± SE for three biological replicates (***P* < 0.01, Student's *t*-test).

TRV:NPR1
CGCCGAAGGACAGGTTGTGTATAGAGATACTAGAGCAAGCTGAAAGAAGAGATCCATT
GCATGGTGAAGCTTCTTTGTCTCTTGCCATCGCTGGTGATGATCTTCGGATGAAGTTGT
TGTATCTCGAAAATAGAGTTGGGCTAGCGAAACTTTTATTCCCAATGGAAGCTAAAGTT
GTGATGGATATAGCTCAAGTGGATGGAACATCGGAGTTCACATTTGCTACCATCAATTC
CAATAAATTAAATGGTGCTCAAACAACAGTGGACTTGAATGAGGCACCTTTCAGGATT
CAAGAGGAGCATTTAAATAGACTCAAAGCACTTTCCAGAACAGTGGAACTCGGGAAG
CGATTTTTTCCTCGTTGCTCCGAAGTGTTGAACAAGATCATGGACGCGGACGACCTATC
GCAGCTAGCTTGCGGAGGGATTGATACCG
TRV:ICS1
TGCCCGTAGCAGCAGGATTTTAACTGCTACAAATATCGATCCTATAGCTTGGTTGGCTTG
TCTGCAGGTTGAAGGAGAAGATGCATATCAGTTTTGTCTTCAGCCACCTAATGGACCCG
CATTTGTGGGAAATACACCAGAGCGACTATTTCACAGAAAATGGCTAAGCATTAGTAGT
GAGGCACTGGCTGCAACCCGCGCTAGAGGTGAATCCAGTGATCTTGATCTTCAAATAG
AGCATGATCTTCTTTCCAGTCCCAAGGACCACCTGGAATTTACCGTAGTACGAGAAAA
CATACAAAACAAATTAGAGTCTGTATGTGATAGAGTTGTTGTTGAACCAAAGAAAACG
GTACGAAAACTTCGAAGAATTCAACATTTATATGCCCAGTTGTCTGGTAACCTGAGAAG
GGAAGATGATGAGTTTGAAATCTTGTCTTCTCTCCACCCAACTCCAGCAGTTT
TRV:COI1
TTTGAAGCTTGATAAATGCTCTGGTTTCTCCACTGATGGACTCTTCCTCATTGGAAGCAT
GTGCCGGCAATTAAGAACCTTGTTTCTTGAAGAGAGTTCAATTGTCGAGAAAGATGGT
CGATGGCTTCACGAGATTGCGGTAAAAAACTCTGTTCTCGAGACTTTAAACTTTTACAT
GACCGATCTTGTCCAAGTGAGTTTCGATGACCTTGAACGGATTGCCAAAAATTGTCCC
AACTTGACCTCGGTGAAAATTAGTGATTGTGAAATTCTGAATCTTGCTGGCTTCTTTCG
TGCTGCTGCCGCTTTAGAAGAATTCTGTGGTGGTTCTTTCAATGAACAATCGGAGAGGT
ATAATGCCGTAAAATTCCCCCCAAGGCTATGCCGTTTGGGTTTAACA

Figure S6 Fragments for construction of the *pTRV2* vectors.

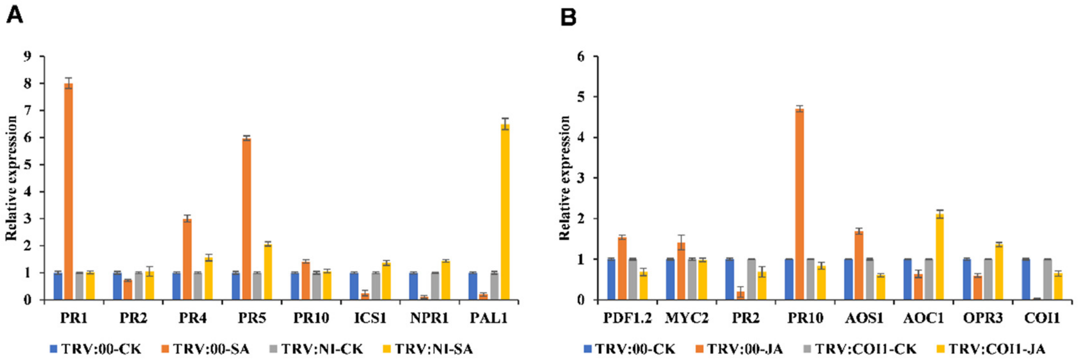

Figure S7 *TRV:NI* and *TRV:COI* plants respectively impaired the SA and JA signaling pathway. **(A)** *TRV:NI* plants impaired in the inducement of *PR* and down-regulation of SA signaling pathway gene expression by the SA treatment. Values represent the means ± SE for three biological replicates. **(B)** *TRV:COI* plants impaired in the inducement of *PR* and down-regulation of JA signaling pathway gene expression by the JA treatment. Values represent the means ± SE for three biological replicates.

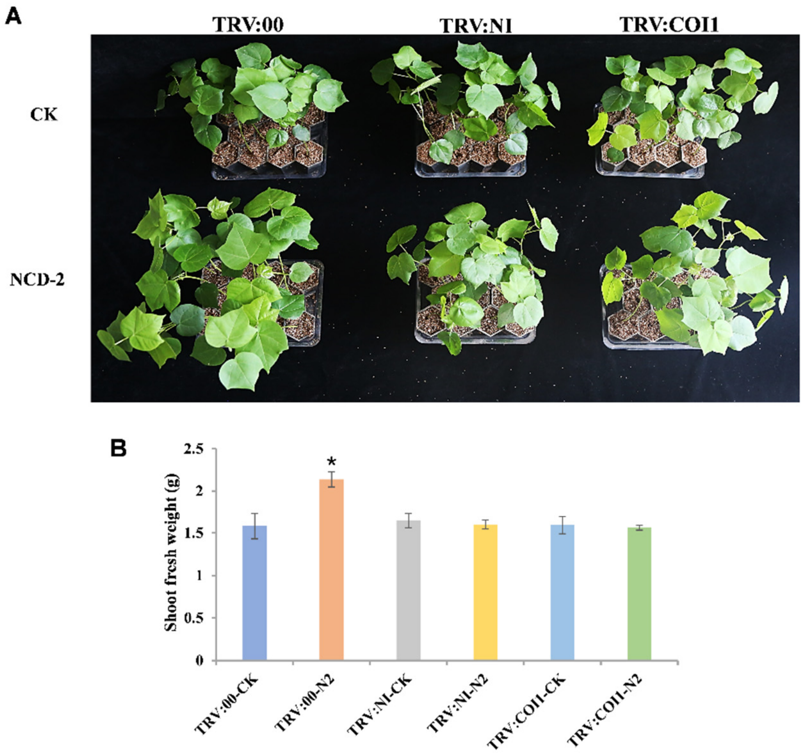

Figure S8 SA and JA signaling pathways are essential for NCD-2-mediated plant growth promotion effect. **(A)** NCD-2 promotes the growth of control plants but not the SA and JA signaling pathways impaired plants. Photographs were taken 20 days post NCD-2 inoculation. **(B)** Shoot fresh weight of the corresponding VIGS plants after NCD-2 treatment.
